# Fish oil supplementation to rats fed high-fat diet during pregnancy prevents development of impaired insulin sensitivity in male adult offspring

**DOI:** 10.1038/s41598-017-05793-0

**Published:** 2017-07-17

**Authors:** Benjamin B. Albert, Mark H. Vickers, Clint Gray, Clare M. Reynolds, Stephanie A. Segovia, José G. B. Derraik, Manohar L. Garg, David Cameron-Smith, Paul L. Hofman, Wayne S. Cutfield

**Affiliations:** 1 Liggins Institute, University of Auckland, Auckland, New Zealand; 2 Nutraceuticals Research Group, School of Biomedical Sciences and Pharmacy, University of Newcastle, Callaghan, NSW Australia; 3 A Better Start – National Science Challenge, University of Auckland, Auckland, New Zealand

## Abstract

We examined whether maternal fish oil supplementation during pregnancy could prevent development of insulin resistance in adult male offspring of rat dams fed a high-fat diet. Time-mated Sprague-Dawley rat dams were randomised into four treatment groups: Con-Con, dams fed a control diet (fat: 15% kcal) and administered water by gavage; Con-FO, control diet with unoxidised fish oil by gavage; HF-Con, high-fat diet (fat: 45% kcal) and water by gavage; and HF-FO, high-fat diet and unoxidised fish oil by gavage. Dams were fed the allocated diet *ad libitum* during pregnancy and lactation, but daily gavage occurred only during pregnancy. After weaning, male offspring consumed a chow diet *ad libitum* until adulthood. Maternal high-fat diet led to increased food consumption, adiposity, systolic blood pressure, and triglycerides and plasma leptin in adult HF-Con offspring. HF-Con offspring also exhibited lower insulin sensitivity than Con-Con rats. Male offspring from HF-FO group were similar to HF-Con regarding food consumption and most metabolic parameters. However, insulin sensitivity in the HF-FO group was improved relative to the HF-Con offspring. Supplementation with unoxidised n-3 PUFA rich oils in the setting of a maternal obesogenic diet improved insulin sensitivity, but had no impact on body composition of adult male offspring.

## Introduction

There has been a dramatic increase in the incidence of obesity and overweight worldwide^1^. As a result, in western countries up to 60% of women of reproductive age are either overweight or obese^2, 3^, and gestational diabetes affects up to 12% of pregnancies^4^.

Maternal pre-pregnancy obesity is associated with adverse changes in body composition in the offspring including macrosomia (and occasionally intrauterine growth retardation), greater adiposity in childhood, and obesity in later life, thus perpetuating a vicious cycle of metabolic disorders across generations^5, 6^. Maternal obesity appears to be the strongest predictor of offspring obesity^7^, and even outside the obese range increasing pre-pregnancy body mass index is associated with greater adiposity and an adverse metabolic phenotype in the offspring^8^. This includes lower insulin sensitivity, an unfavourable adipokine profile, increased blood pressure and triglyceride concentrations^9, 10^ and increased risk of type 2 diabetes and the metabolic syndrome^11^. While the mechanisms remain unclear, the adverse effects seen in the offspring appear to be mediated (at least in part) by exaggeration of the normal insulin resistance of pregnancy. This leads to excess delivery of glucose and fatty acids to the fetus^12^, and ultimately to lifelong alterations in gene expression through epigenetic changes^13^.

Adverse metabolic programming of offspring as a consequence of a maternal obesogenic environment has important implications for our increasingly overweight society. The origins of the obesity epidemic may lie in a postnatal environment characterized by abundance of high-energy foods and an increasingly sedentary lifestyle. However, now that obesity is common, a second factor has emerged: the transgenerational perpetuation of obesity and metabolic disease through maternal obesity and insulin resistance^14^. This highlights pregnancy as a critical early window of opportunity where treatments that reduce fetal overnutrition may have lifelong benefit^14^, potentially improving insulin sensitivity and in turn reducing the risk of type 2 diabetes and cardiovascular disease^15^.

In rodent pregnancy, insulin resistance is readily induced by a high-fat diet rich in saturated fat^16–18^, which also leads to an offspring phenotype of leptin resistance with hyperphagia^19, 20^, increased body weight and adiposity^16, 17, 20^, reduced insulin sensitivity^16, 20^, dyslipidaemia^20^, and elevated blood pressure^17, 20^. This phenotype is similar to the offspring of insulin resistant women^8, 11^. The long-term metabolic effects of altering maternal consumption of polyunsaturated fatty acids are not well defined. However, studies have shown that: (a) greater consumption of omega-6 polyunsaturated fatty acids (n-6 PUFAs) is associated with greater adiposity in the offspring^21^, (b) increasing omega-3 (n-3) PUFA consumption may reduce offspring adiposity^22^, and (c) a diet deficient in n-3 PUFAs leads to abnormalities of appetite regulation^23^. The ratio of n-6:n-3 PUFAs also appears to be important, and this is probably because n-3 and n-6 PUFAs compete at key enzymes for elongation and production of active metabolites^24^.

n-3 PUFAs have anti-inflammatory and insulin-sensitising effects that could make them a useful treatment to prevent the long-term effects of maternal insulin resistance in the offspring. In non-pregnant rodents, insulin resistance can be prevented by supplementation with fish oil^25, 26^, an effect mediated by the anti-inflammatory effects of n-3 PUFAs in adipose tissue^25^. Recent evidence suggests that n-3 PUFA supplementation is also insulin-sensitising in humans^27, 28^, and that supplementation in obese pregnant women reduces adipose tissue inflammation^29^. These observations raise the possibility that fish oil supplementation in pregnancy could prevent metabolic dysfunction in the offspring of insulin-resistant women. Thus, we utilised an established rat model of high-fat feeding in pregnancy to test the primary hypothesis that maternal fish oil supplementation in pregnancy would prevent the development of insulin resistance in the offspring. In addition, we aimed to assess the effects of the fish oil intervention on body composition and other metabolic markers in the adult offspring.

## Methods

### Animal Ethics

Ethics approval was granted by the Auckland University Animal Ethics Committee (approval #001175). This study was performed in accordance with all appropriate institutional and international guidelines and regulations for animal research.

### Study design

**Figure 1:**
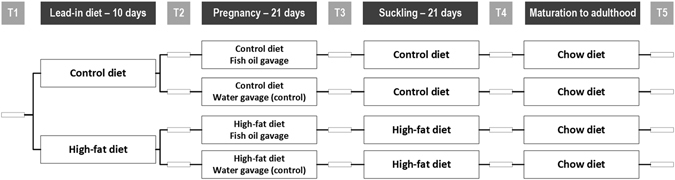
Diagram describing individual steps of the trial and indicating the timing of assessments. T1: Virgin female rats were randomised to control or high-fat diet. T2: Females were mated and further randomised to one of the two gavage treatments, i.e. Fish oil or Control (water). T3: on postnatal day 2 pups were counted, sexed and weighed; excess pups were culled. T4: on postnatal day 21 pups were separated, while dams were culled and had samples taken. T5: final metabolic assessments of adult male offspring.

A model of maternal obesity was utilised as previously reported by our group, in which a maternal high-fat diet leads to increased body fat in the offspring and results in fasting hyperinsulinaemia and hyperleptinaemia (compared with rats exposed to a standard maternal diet), independently of postnatal diet^16^. Fifty-two virgin female Sprague-Dawley rats (age 110 days) were assigned to one of two isocaloric diets *ad libitum* for ten days prior to mating: a high-fat diet (HF, D12451, Research Diets Inc, New Brunswick, NJ USA) which contained 45% kcal as fat, or a matched control diet (D12450H) containing 10% kcal as fat (Table 1). Rats were time-mated using an oestrous cycle monitor (EC-40, Fine Science Tools, San Francisco, CA, USA). Day 1 of pregnancy was determined by detection of spermatozoa by vaginal lavage, and dams were individually housed and continued on their respective study diet. On Day 1 of pregnancy, dams were further allocated to one of two treatment groups: fish oil (FO) or water (control). On each day of pregnancy, 1 ml of the treatment was administered by gavage. Thus, there were four treatment groups: (1) Con-Con, where dams consumed a control diet and had water by gavage; (2) Con-FO, where dams consumed a control diet and had fish oil by gavage; (3) HF-Con, where dams consumed a high-fat diet and had water by gavage; and (4) HF-FO, where dams had high-fat diet and fish oil by gavage (Fig. 1). Importantly, as dams from all groups underwent daily gavage, any potential stressful effects related to the gavage procedure were controlled for.

**Table 1:** Nutritional content of control and high-fat diets given to rat dams during pregnancy and lactation.

Macronutrient	Description	Unit	Control diet (D12450H)	High-fat diet (D12451)
Protein	Total	% kcal	20	20
Carbohydrate	Sucrose	% kcal	17	17
	Other carbohydrates	% kcal	53	18
Fat	Total	% kcal	10	45
	Saturated fats	% of total fat (wt)	22.7	31.4
	Monounsaturated fats	% of total fat (wt)	29.9	35.5
	Polyunsaturated fats	% of total fat (wt)	47.4	33.1
	Eicosapentaenoic acid (EPA)	% of total fat (wt)	0	0
	Docosahexaenoic acid (DHA)	% of total fat (wt)	0	0

Data have been provided by the manufacturer (Research Diets Inc.).

The treatment oil was derived from hoki (*Macruronus novaezelandiae*) liver (Seadragon Marine Oils, Nelson, New Zealand). We independently verified n-3 PUFA content (Table 2) and the oxidative state, using methodology previously described^30^. The oil had very low levels of oxidation, with a peroxide value of 2.7 meq/kg and anisidine value of 0.6 at the start of the study, well within international recommendations^31^ and below all products tested in a recent study of fish oil products available for purchase in the New Zealand market^30^. At the end of the study, the oil remained unoxidised, with a peroxide value of 3.1 meq/kg and ansidine value of 0.9. One ml/day of fish oil equated to a daily dose of 39 mg eicosapentaenoic acid (EPA) and 82 mg docosahexaenoic acid (DHA). To ensure standardised dosage and to prevent inadvertent oxidation, delivery of the treatment (oil or water) was administered by oral gavage. In addition, the fish oil was stored in small aliquots, frozen, sealed, and kept in darkness, so that each day a new aliquot was thawed prior to use, minimizing any oxidation of the oil. Minimising the risk of oxidation is important, as in a parallel study we demonstrated that feeding highly oxidised fish oil (peroxide value 48.8 meq/kg, anisidine value 4.5) to rat dams led to maternal insulin resistance and a substantial increase in neonatal mortality^32^.

**Table 2:** Fatty acid concentrations in the fish oil supplement (mg/g of oil), determined by gas chromatography.

Fatty acid	Concentration
C16:0 (palmitic acid)	105.53 ± 4.92
C16:1n-7 (palmitoleic acid)	15.86 ± 0.84
C18:0 (stearic acid)	28.26 ± 1.37
C18:1n-7 (cis-vaccenic acid)	4.35 ± 0.12
C18:1n-9 (oleic acid)	425.59 ± 19.58
C18:2n-6 (linoleic acid)	3.12 ± 0.09
C18:3n-3 (α-linolenic acid)	5.52 ± 0.13
C18:3n-6 (γ-linolenic acid)	1.68 ± 0.59
C20:0 (arachidic acid)	0.72 ± 0.03
C20:1n-9 (eicosenoic acid)	0.47 ± 0.03
C20:2n-6 (eicosadienoic acid)	1.67 ± 0.08
C20:3n-6 (dihomo-γ-linolenic acid)	4.10 ± 0.27
C20:4n-6 (arachidonic acid)	2.90 ± 0.19
C20:5n-3 (eicosapentaenoic acid)	42.53 ± 1.85
C22:5n-3 (docosapentaenoic acid)	1.09 ± 0.08
C22:6n-3 (docosahexaenoic acid)	89.00 ± 4.34

Data are means ± standard deviations, from analysis of 6 replicates.

Maternal food intake and body weight were recorded every 3^rd^ day. At birth, the gavage treatment was stopped, but the same diets were maintained *ad libitum* throughout lactation. Offspring were counted, sexed (by measuring anogenital distance), weighed and measured on postnatal day 2. On this day, litter size was also randomly adjusted to 8 pups to ensure standardized nutrition until weaning. The primary aim of this study was to assess metabolic phenotype in adult male offspring to avoid the confounding effect of oestrous on insulin sensitivity, thus 6 males and 2 females were kept. Pups not allocated to litters were killed by decapitation, when plasma, red blood cells, liver, and heart were collected. Pups were weighed every 3^rd^ day until the time of weaning (day 21), when they were housed in pairs for the remainder of the study (until day 110). All offspring were fed a standard chow diet (Harlan-Teklad Diet 2018, Oxon, UK) *ad libitum*, with food intakes and body weights recorded every 3^rd^ day.

From the adult offspring within each of the 4 treatment groups, 12 animals were randomly selected for metabolic assessment.

At postnatal day 90, body composition was quantified using dual x-ray absorptiometry (DXA, Lunar Prodigy 2000, General Electric, Madison, USA), while animals were under light isoflurane anaesthesia.

Systolic blood pressure was analysed at 95 days of age by tail cuff plethysmography (Model 179 with an automatic cuff inflation pump (NW20), IITC, Life Science, Woodland Hills, CA), according to the manufacturer’s instructions as previously described^33^. Adult offspring were warmed using a heat lamp and acclimatised to the restraint tube for 15 minutes prior to blood pressure recordings. Three clear pressure recordings were taken per animal (CV < 5%) and mean blood pressure calculated.

At day 100, insulin sensitivity was assessed by an oral glucose tolerance test following a 6-hour fast. After collection of tail blood (at T0), a glucose load (1 g/kg of body weight) was given by gavage. Blood was collected from the tail at T30, T60, T90 and T120 minutes. Glucose was measured at each time point, with plasma insulin also measured at T0, T30, T60, T90 and T120. Insulin and glucose concentrations were used to derive the Matsuda index of insulin sensitivity^34^, which was the primary outcome of this study. The Matsuda index of insulin sensitivity has been shown to be closely correlated to the hyperinsulinaemic euglycaemic clamp (r = 0.77)^35^. Its use in rats has been recently described^36^. In addition, we also calculated HOMA-IR, which is a fasting index of insulin resistance well correlated to the hyperinsulinaemic euglycaemic clamp (r = 0.7)^37^ and widely reported in rats, as well as glucose and insulin area under the curve (AUC) using the trapezoidal method.

### Terminal sample collection

At day 110, adult offspring were fasted overnight and killed by sodium pentobarbitone anaesthesia (60 mg/kg; intraperitoneal) followed by decapitation. Blood was collected in EDTA tubes and stored on ice until centrifugation and removal of plasma for analysis. Liver and the retroperitoneal and gonadal fat pads were harvested and weighed.

### Assays

During the oral glucose tolerance test, glucose concentration was measured by glucometer (Optium Xceed; Abbott Laboratories) from tail whole blood, at the time samples were taken. Insulin and leptin concentrations were measured by ELISA (Crystal Chem, Illinois, USA) with CVs of 11% and 7%, respectively. Glucose, creatinine, alkaline phosphatase (ALP), alanine transaminase (ALT), aspartate transaminase (AST), free fatty acids, uric acid, triglycerides, high-density lipoprotein cholesterol (HDL-C), low-density lipoprotein cholesterol (LDL-C), and total cholesterol were measured on a Hitachi 902 autoanalyser (Hitachi High Technologies Corporation, Tokyo, Japan) with all CVs less than 6%.

### Primary outcome and power analysis

The primary outcome of this study was the Matsuda index of insulin sensitivity in the adult male offspring. Two major comparisons were required: 1) HF-Con vs Con-Con to show the effect of the high-fat diet in pregnancy and lactation, and 2) HF-FO vs HF-Con to show the additional effect of further supplementation with fish oil to dams consuming a high-fat diet. Due to limited data regarding the standard deviation of the Matsuda index in rats, sample size was calculated to enable differences to be shown in fasting insulin. Given previous data showing maternal high-fat diet was associated with a mean fasting insulin of 4.89 ng/ml and standard deviation of 0.926 in the adult male offspring^16^, a sample of approximately 12 animals per group was required to have 90% power to detect a 25% change in fasting insulin.

### Statistical analyses

Outcomes in rat dam were compared using general linear regression models, with diet and gavage included as factors. Offspring outcomes were assessed using linear mixed models, including dam identification code as a random factor. The interaction between diet and gavage was examined in all models. Differences in weight, food and energy consumption in dams and offspring over time were compared using multiple t tests and Holm-Sidak correction for multiple comparisons. All statistical analyses were carried out in SAS v.9.4 (SAS Institute, Cary, NC, USA), except t tests which were carried out in Prism v.6.03 (Graphpad Software Inc, La Jolla, CA, USA). All tests were two-tailed with significance level maintained at 5%.

## Results

Of the 52 rat dams mated, 34 became pregnant and successfully had pups (Table 3). Neither diet, nor gavage treatment affected the number of offspring per litter (Table 3).

**Table 3:** Maternal weight and metabolic parameters at key milestones of the study and litter characteristics at birth, according to the maternal diet (control or high-fat) and the gavage treatment during pregnancy [fish oil or control (water)].

	Maternal control diet		Maternal high-fat diet	
	Control	Fish oil	Control	Fish oil
n	8	9	9	8
Maternal weight (g)				
Prior to diet allocation (day -10)	301 ± 6	298 ± 10	296 ± 5	303 ± 6
Mating (day 1)	310 ± 7	307 ± 10	322 ± 7	332 ± 12
End of pregnancy (day 22)	434 ± 6	444 ± 14	457 ± 16	445 ± 17
End of lactation (day 40)	342 ± 4	359 ± 13	368 ± 9^†^	360 ± 11
Gestational weight gain	40 ± 2	45 ± 2	42 ± 4	34 ± 3
Retroperitoneal fat (% of body weight at day 40)	1.2 ± 0.2	1.3 ± 0.2	1.6 ± 0.1^†^	1.2 ± 0.1^#^
Metabolic parameters (day 40)				
Fasting glucose (mmol/l)	4.44 ± 0.17	4.56 ± 0.15	5.20 ± 0.32^†^	4.93 ± 0.18
Fasting insulin (pmol/l)	180 ± 19	323 ± 118	403 ± 91	332 ± 58
HOMA-IR	0.98 ± 0.09	1.84 ± 0.66	2.58 ± 0.58^‡^	2.01 ± 0.37
Triglycerides (mmol/l)	0.52 ± 0.03	0.47 ± 0.02	0.68 ± 0.05^†^	0.72 ± 0.05
Birth characteristics				
Litter size (n)	11.8 ± 1.0	14.1 ± 0.9	13.7 ± 0.8	11.9 ± 1.1
Sex ratio (% male per litter)	45 ± 5	42 ± 4	57 ± 3^†^	50 ± 4
Weight of pups (g)	5.6 ± 0.3	5.6 ± 0.3	5.4 ± 0.2	6.2 ± 0.4*
Length of pups (mm)	47.7 ± 0.2	47.4 ± 0.2	46.7 ± 0.4	48.9 ± 0.2**

Data are means ± standard errors. ^‡^p < 0.06 and ^†^p < 0.05 for a maternal diet effect among controls; ^#^p < 0.06, *p < 0.05, and **p < 0.01 for an effect of the gavage treatment between groups exposed to the same maternal diet.

### Effects of maternal high-fat diet

**Figure 2:**
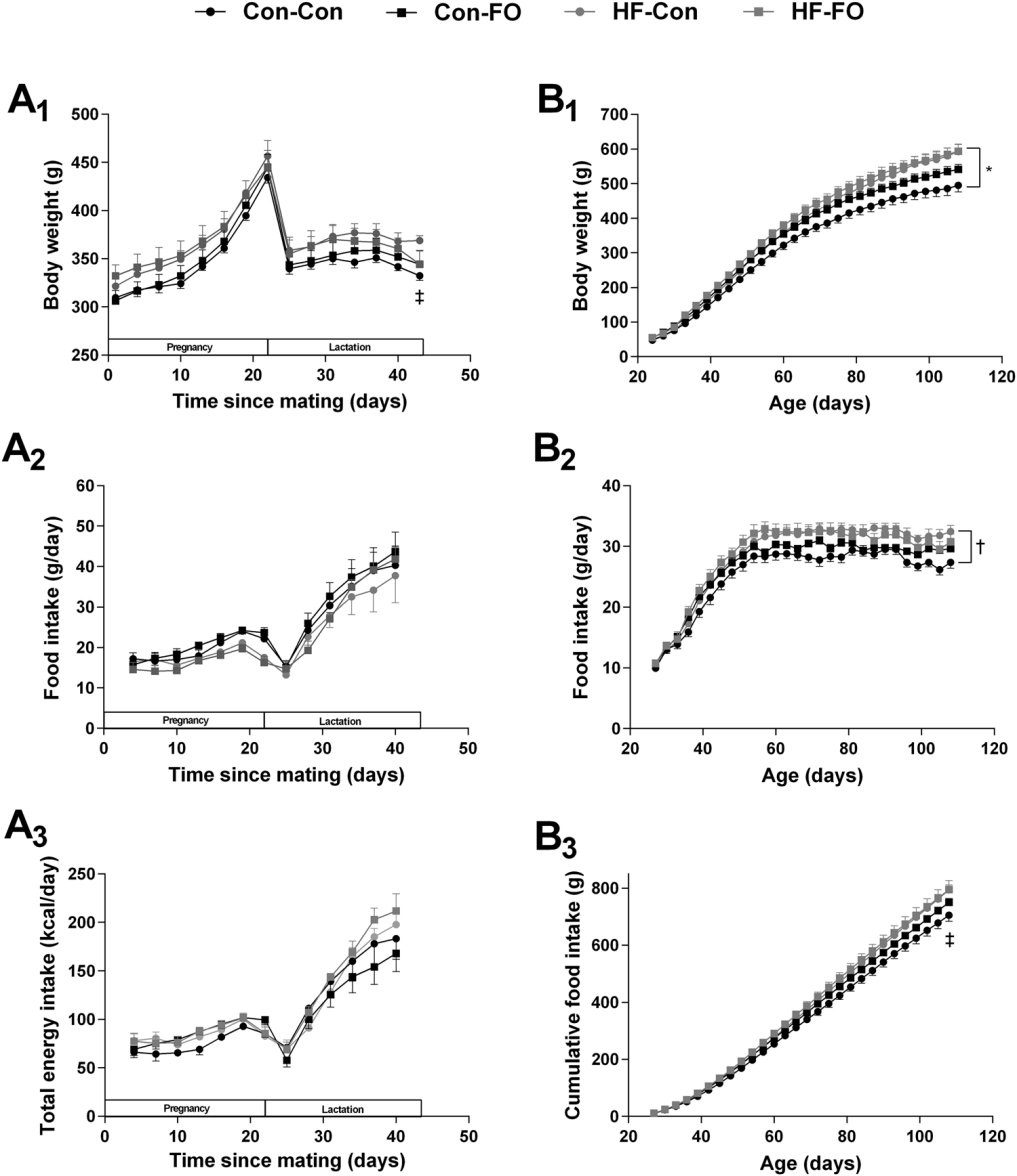
Weight, food consumption, and energy intake in rat dams (**A**) and male offspring (**B**) according to the maternal diet (control or high-fat) and gavaged treatment during pregnancy [fish oil or control (water)]. A: body weight (A1), food consumption (A2), and total energy consumption including diet and gavaged oil (A3) of dams from mating (day 0) through to weaning (day 40). B: body weight (B1), food consumption (B2), and cumulative food consumption of male offspring from weaning through to termination (day 110) (B3). Error bars represent standard errors; *p < 0.05 for a difference in weight between Con-Con and HF-Con groups across the life span; ^†^p < 0.05 for a difference in food intake between Con-Con and HF-Con groups from day 60 when intake stabilized; ^‡^p < 0.05 for a difference between Con-Con and HF-Con groups at termination.

At the end of lactation, dams from the HF-Con group had similar energy intake to Con-Con dams, but had greater body weight (Fig. 2A) and retroperitoneal fat mass (Table 3). This was associated with greater fasting glucose (p = 0.018) and triglyceride (p = 0.047) concentrations, as well as a trend to greater insulin resistance (i.e. higher HOMA-IR; p = 0.058) (Table 3).

HF-Con litters had a greater proportion of male pups than the Con-Con group (Table 3). Male HF-Con offspring had greater food consumption and body weight (than in the Con-Con group), which were first detectable at postnatal day 57 (Fig. 2B). In the final assessments, adult males of the HF-Con group had greater body weight (+98 g; p = 0.030) and adiposity (+6.4% of body fat; p = 0.008), with proportionally greater retroperitoneal (+1.11%; p = 0.009) and gonadal (+0.63%; p = 0.002) fat compared with Con-Con males (Table 4).

**Table 4:** Study outcomes among male offspring according to the maternal diet (control or high-fat) and the gavage treatment during pregnancy [fish oil or control (water)].

	Time		Maternal control diet		Maternal high-fat diet	
			Control	Fish oil	Control	Fish oil
n			12	12	12	12
Pubertal onset (days)			43.3 ± 0.6	41.3 ± 0.6	43.4 ± 0.6	42.2 ± 0.5
Auxology	24 d	Weaning weight (g)	48 ± 3	54 ± 2	54 ± 3	61 ± 3
	90 d	Total body fat (%)	22.9 ± 1.1	24.2 ± 1.2	29.3 ± 1.0^††^	27.0 ± 1.4
		Fat mass to lean mass ratio	0.30 ± 0.02	0.32 ± 0.02	0.42 ± 0.02^††^	0.38 ± 0.03
		Bone mineral density (g/cm^2^)	0.154 ± 0.002	0.156 ± 0.002	0.158 ± 0.002	0.161 ± 0.002
	108 d	Weight (g)	495 ± 19	541 ± 15	593 ± 22^†^	593 ± 19
		Length (mm)	258 ± 2	258 ± 2	266 ± 3	262 ± 2
	110 d	Liver weight (% body weight)	2.85 ± 0.09	3.01 ± 0.06	3.01 ± 0.09	2.97 ± 0.07
		Gonadal fat (% body weight)	1.77 ± 0.011	2.15 ± 0.12	2.40 ± 0.13^††^	2.27 ± 0.18
		Retroperitoneal fat (% body weight)	1.98 ± 0.30	2.31 ± 0.16	3.09 ± 0.25^††^	2.68 ± 0.21
Diet	108 d	Energy intake (kcal/day)	85 ± 3	92 ± 2	101 ± 3^†^	95 ± 4
		Energy intake per body weight (kcal/g/day)	0.17 ± 0.01	0.17 ± 0.01	0.17 ± 0.01	0.16 ± 0.01
		Cumulative food intake (g)	705 ± 22	751 ± 13	794 ± 17^†^	795 ± 31

“Cumulative food intake” reflects the weight of all food consumed by an individual rat since weaning. Data are means ± standard errors. ^†^p < 0.05 and ^††^p < 0.01 for a maternal diet effect among the offspring of dams that received control gavage. Note that all study groups consisted of 5 to 7 separate litters. Time represents the age of the animals in days.

Importantly, the male adult HF-Con offspring had lower insulin sensitivity than the Con-Con rats, as indicated by a lower Matsuda index (−38%; p = 0.036), higher HOMA-IR (+51%; p = 0.042), and greater insulin AUC (+63%; p = 0.023) (Table 5). This was accompanied by other markers of metabolic dysfunction, including higher systolic blood pressure (+12 mmHg, p < 0.0001), and increased triglyceride (+0.55 mmol/l; p = 0.014) and leptin (+4.1 ng/ml; p = 0.002) concentrations (Table 5). Thus, as expected^16–20^, maternal high-fat diet led to adverse programming of energy balance, body composition, and metabolism in adult male offspring.

**Table 5:** Study outcomes among adult male offspring according to the maternal diet (control or high-fat), and gavage treatment during pregnancy [fish oil or control (water)].

	Maternal control diet		Maternal high-fat diet	
	Control	Fish oil	Control	Fish oil
n	12	12	12	12
Glucose homeostasis				
Matsuda index	1.35 ± 0.17	1.27 ± 0.25	0.84 ± 0.09^†^	1.55 ± 0.26*
HOMA-IR	3.02 ± 0.41	3.89 ± 0.63	4.57 ± 0.54^†^	2.66 ± 0.42*
Fasting glucose (mmol/l)	4.96 ± 0.14	5.46 ± 0.21	5.03 ± 0.11	5.03 ± 0.21
Glucose AUC (min.mmol/l)	847 ± 25	841 ± 28	866 ± 31	818 ± 29
Fasting insulin (pmol/l)	497 ± 63	556 ± 74	732 ± 79	434 ± 71*
Insulin AUC (x10^3^ min.pmol/l)	76 ± 9	82 ± 11	124 ± 14^†^	81 ± 9
Liver function				
ALP (U/l)	79 ± 4	66 ± 3	76 ± 5	82 ± 6
AST (U/l)	144 ± 13	133 ± 5	171 ± 9	160 ± 14
ALT (U/l)	36 ± 2	35 ± 2	38 ± 4	38 ± 2
Lipids				
Free fatty acids (mmol/l)	0.83 ± 0.09	0.79 ± 0.07	0.95 ± 0.12	0.83 ± 0.08
Total cholesterol (mmol/l)	1.70 ± 0.13	1.94 ± 0.11	1.96 ± 0.15	1.81 ± 0.09
HDL-C (mmol/l)	1.21 ± 0.09	1.46 ± 0.08	1.37 ± 0.10	1.39 ± 0.06
LDL-C (mmol/l)	0.29 ± 0.03	0.25 ± 0.03	0.25 ± 0.02	0.25 ± 0.03
Triglycerides (mmol/l)	0.68 ± 0.07	0.94 ± 0.09	1.23 ± 0.15^†^	0.87 ± 0.11
Other				
Uric acid (µmol/l)	71 ± 5	75 ± 2	73 ± 3	69 ± 6
Leptin (ng/ml)	1.6 ± 0.3	4.2 ± 1.3*	5.7 ± 1.3^††^	4.0 ± 0.9
Systolic blood pressure (mmHg)	124 ± 1	125 ± 1	136 ± 1^††††^	135 ± 1

Data are means ± standard errors. ^†^p < 0.05, ^††^p < 0.01, and ^††††^p < 0.0001 for a maternal diet effect among the offspring of dams that received control gavage; *p < 0.05 and **p < 0.01 for an effect of the gavage treatment between groups exposed to the same maternal diet. ALP – alkaline phosphatase; ALT – alanine transaminase; AST – aspartate transaminase; AUC – area under the curve; HDL-C – high-density lipoprotein cholesterol; and LDL-C low-density lipoprotein cholesterol.

### Effects of supplemental fish oil in the context of a maternal high-fat diet

HF-FO dams were not different from HF-Con dams in terms of body weight, food intake, or total energy intake (Fig. 2A). There were no effects of fish oil supplementation on metabolic parameters measured (Table 3). However, HF-FO pups were heavier and longer than HF-Con pups (Table 3), and had 33% greater EPA concentration in liver phospholipid (Table 6).

**Table 6:** Concentrations of polyunsaturated fatty acids in liver phospholipids of rat pups culled at day 2, according to the maternal diet (control or high-fat) and the gavage treatment during pregnancy [fish oil or control (water)].

	Maternal control diet		Maternal high-fat diet	
	Control	Fish oil	Control	Fish oil
n	9	12	17	8
C16:0 (palmitic acid)	24.49 ± 1.06	24.41 ± 1.08	23.21 ± 0.58	24.13 ± 0.50
C16:1n-7 (palmitoleic acid)	1.32 ± 0.09	1.26 ± 0.07	0.70 ± 0.05^††††^	0.65 ± 0.05
C18:0 (stearic acid)	16.61 ± 1.04	16.19 ± 0.88	17.06 ± 0.61	15.53 ± 0.86
C18:1n-7 (cis-vaccenic acid)	1.87 ± 0.16	1.95 ± 0.11	1.61 ± 0.10	1.33 ± 0.08
C18:1n-9 (oleic acid)	9.67 ± 0.58	9.60 ± 0.48	10.47 ± 0.63	9.47 ± 1.00
C18:2n-6 (linoleic acid)	10.74 ± 0.40	10.45 ± 0.65	11.13 ± 0.35	11.39 ± 0.39
C18:3n-3 (α-linolenic acid)	0.23 ± 0.04	0.25 ± 0.03	0.19 ± 0.02	0.24 ± 0.02
C18:3n-6 (γ-linolenic acid)	0.44 ± 0.06	0.35 ± 0.03	0.36 ± 0.03	0.43 ± 0.048
C20:0 (arachidic acid)	0.62 ± 0.07	0.52 ± 0.06	0.60 ± 0.05	0.61 ± 0.06
C20:1n-9 (eicosenoic acid)	0.21 ± 0.03	0.28 ± 0.03	0.20 ± 0.01	0.26 ± 0.03
C20:2n-6 (eicosadienoic acid)	0.73 ± 0.05	0.72 ± 0.04	0.60 ± 0.03^†^	0.61 ± 0.06
C20:3n-6 (dihomo-γ-linolenic acid)	0.75 ± 0.09	0.71 ± 0.06	0.85 ± 0.06	0.81 ± 0.10
C20:4n-6 (arachidonic acid)	17.57 ± 0.97	18.57 ± 1.25	18.84 ± 0.76	18.42 ± 0.60
C20:5n-3 (eicosapentaenoic acid)	0.65 ± 0.07	0.66 ± 0.05	0.51 ± 0.04	0.68 ± 0.05**
C22:4n-6 (adrenic acid)	4.06 ± 0.57	3.56 ± 0.46	3.37 ± 0.29	4.21 ± 0.23
C22:5n-6 (n-6 docosapentaenoic acid)	1.46 ± 0.29	1.39 ± 0.16	1.27 ± 0.11	0.19 ± 0.13
C22:5n-3 (n-3 docosapentaenoic acid)	4.71 ± 0.31	5.03 ± 0.28	4.77 ± 0.27	5.82 ± 0.53
C22:6n-3 (docosahexaenoic acid)	3.87 ± 0.33	4.18 ± 0.44	4.29 ± 0.25	4.23 ± 0.36

Data are means ± standard errors. ^†^p < 0.05 and ^††††^p < 0.0001 for a maternal diet effect among the offspring of dams that received control gavage; **p < 0.01 for an effect of the gavage treatment between groups exposed to the same maternal diet.

Adult male offspring from the HF-FO group were no different from HF-Con in terms of food consumption, body weight (Fig. 2B), adiposity, or proportional liver weight (Table 4). However, insulin sensitivity in the HF-FO group was greater than in the HF-Con group as indicated by higher Matsuda index (+85%; p = 0.014), lower HOMA-IR (−42%; p = 0.021), lower fasting insulin concentrations (−41%; p = 0.026), and a trend towards reduced insulin AUC (−35%; p = 0.052) (Table 5). Of note, HF-FO indices of insulin sensitivity were similar to those in the Con-Con group (Table 5) (p = 0.48 and p = 0.36 for Matsuda index and HOMA-IR, respectively). However, there were no differences in blood pressure, triglycerides, or leptin in adult male rats from HF-FO and HF-Con groups (Table 5).

### Effects of supplemental fish oil in control diet

There were no differences between the Con-FO and the Con-Con group in terms of food consumption, body weight, adiposity, insulin sensitivity, lipid profile, or systolic blood pressure (Tables 4 and 5). However, leptin levels were 2.6 times higher in adult Con-FO males (p = 0.027; Table 5), an effect that remained after controlling for body fat percentage (p = 0.045).

## Discussion

In this study, a rodent model of maternal high-fat feeding was used, which is known to induce maternal insulin resistance^18^ and metabolic abnormalities in the offspring^16, 18, 20^. As expected^16–20^, despite a standard postnatal diet the adult male offspring developed obesity associated with increased caloric intake, as well as reduced insulin sensitivity, increased plasma leptin and triglyceride levels, and higher systolic blood pressure. Importantly, maternal fish oil supplementation during pregnancy prevented the impairment of insulin sensitivity in the offspring. Given the established relationship between insulin sensitivity, type 2 diabetes mellitus, and the cardiovascular risk factors that make up the metabolic syndrome^15^, supplementation with unoxidised fish oil during pregnancy could be a useful approach to reduce cardio-metabolic risk in the offspring of pregnancies complicated by obesity.

The intention of the fish oil treatment in pregnancy was to indirectly affect the developing pup by improving maternal insulin sensitivity, and therefore reducing excessive transfer of lipid and glucose to the fetus. While we were not able to demonstrate improved insulin sensitivity in the HF-FO dams, this does not indicate that the well-established insulin-sensitising effects of n-3 PUFAs in rodents^25, 26^ do not occur during pregnancy. The metabolic assessment of dams was conducted at the end of lactation, when the dams had continued to consume a high-fat diet, but had not been supplemented with fish oil for 21 days. Thus, potential insulin-sensitising effects of the fish oil treatment would be expected to be lost. Nevertheless, alternative mechanisms are possible. n-3 PUFAs are actively transported across the placenta^38^, so that the n-3 PUFA rich supplement could have direct effects on fetal metabolism. For example, as n-3 PUFAs activate PPAR-γ^39^, which is critical in adipogenesis^40^, it is possible that n-3 PUFAs could have had an important effect on the developing adipose tissue in our study.

Fish oil supplementation in dams fed a control diet did not lead to any improvement in metabolic markers in the adult offspring. Unexpectedly, fasting leptin was elevated in the Con-FO group, even after adjustment for body fat. While hyperleptinaemia is considered unfavourable^41^, it is not clear whether the increased leptin levels observed represent a primary increase in leptin secretion by adipose tissue, or a secondary increase due to leptin resistance. Pathological leptin resistance is unlikely since leptin has an important effect on appetite, and there were no differences in food consumption between groups. However, to determine whether there was subtle leptin resistance would require additional manipulations, such as assessing the effects of leptin injection on appetite suppression or response to a fasting challenge. Nevertheless, although there were no other adverse effects of fish oil supplementation, our study does not support any metabolic benefits of n-3 PUFA supplementation in the context of a maternal control diet, which models normal pregnancy.

While fish oil supplementation prevented the development of insulin resistance in the offspring of dams fed a high-fat diet, it did not prevent other adverse metabolic effects. This divergence of effects on insulin sensitivity and other metabolic parameters suggests that there are important differences in the programming of insulin sensitivity from other key aspects of the metabolic phenotype, specifically body composition, blood pressure, and leptin resistance.

One possible explanation for the lack of effect of the fish oil treatment on offspring outcomes (other than insulin sensitivity) could be that supplementation did not occur during lactation. In rats, a maternal high-fat diet, whether in pregnancy or solely during lactation, leads to greater adiposity and increased leptin, triglycerides, and blood pressure in the adult offspring^16, 17^. Thus, while fish oil ameliorated some of the adverse effects of a maternal high-fat diet during pregnancy, its effect during lactation could have been limited, as supplementation was solely during pregnancy and maternal stores would diminish over time. In this context, it was not surprising that HF-FO offspring displayed a phenotype of greater adiposity, increased leptin and triglyceride levels, and elevated systolic blood pressure compared to Con-Con offspring. We speculate that there would have been benefits of fish oil on energy balance, adiposity, triglycerides, and blood pressure if supplementation could have been continued throughout lactation.

As fish oil contains a range of fatty acids in addition to n-3 PUFAs, the potential effects of other fatty acids in the fish oil should also be considered. Taking into account both diet and supplement, the HF-FO dams consumed 17.5% more oleic acid than the HF-Con group (on day 19 of pregnancy), but intake of other fatty acids was similar. This did not affect the levels of oleic acid in tissue of the neonatal pups. The effects of changes in the intake of dietary oleic acid during pregnancy on the offspring are unknown. However, oleic acid-rich oils are commonly used as control treatments in metabolic studies^42–44^, and oleic acid does not activate the pathway that mediates the pro-adipogenic effects of n-6 PUFAs in pregnancy^24^.

Interestingly, a maternal high-fat diet skewed the sex ratio in the offspring in favour of males, while sex ratio was unaffected by fish oil supplementation. Such skewing has been reported in previous rodent studies examining diets high in saturated fat^45, 46^, fructose^47^, and salt^48^, but the mechanism has not been elucidated.

The maternal high-fat diet model of developmental programming in rodents appears to be relevant to humans. Obesity, and in particular visceral obesity, is associated with adipose tissue inflammation^49, 50^, which leads to insulin resistance through abnormal adipokine secretion and free fatty acid release^51^. Similarly, high-fat diets have been shown to increase inflammation of adipose tissue, through saturated fatty acids binding to toll-like receptor-4^52^. Thus, the obese human condition and the obesogenic rat model are both characterized by adipose tissue inflammation and dysfunction. In addition, women who are obese and pregnant do tend to consume excess saturated fat^53^.

In humans, studies investigating the effects of supplemental fish oil during pregnancy on offspring body composition or metabolism have yielded conflicting results in infancy and childhood^54^ and no effect in young adults^55^. However, these studies did not enrol women likely to have abnormally poor insulin sensitivity. As shown here, unoxidised fish oil supplementation in the context of the control diet (which modelled normal pregnancy) led to no metabolic improvements in the adult offspring. We speculate that that in obese human pregnancies (but not in normal pregnancies) supplementation with n-3 PUFAs (from pure concentrate or unoxidised fish oil) will lead to beneficial effects on body composition and metabolism of the offspring.

This study represents the first investigation into whether fish oil supplementation during pregnancy could prevent metabolic dysfunction in the offspring arising as a consequence of a maternal obesogenic environment. We focused on the metabolic phenotype of the offspring, and not the underlying mechanisms. This was because many of the assessments required to elucidate mechanism would likely affect offspring survival or metabolism (and thus confound the study), so that they would have to be conducted in parallel cohorts that would be culled after assessment (e.g. assessment of insulin sensitivity during pregnancy or collection of milk). However, having identified an important effect of fish oil supplementation on phenotype, such mechanistic studies should now be planned.

Our study has a number of limitations, primarily regarding the lack of data on mechanisms underpinning our observations. For example, as we did not assess maternal insulin sensitivity during pregnancy, we were not able to show whether fish oil supplementation improved maternal glucose metabolism during pregnancy. Similarly, as milk samples were not collected from rat dams, we could not determine whether pup development was influenced by possible changes in milk composition. Additional limitations include the focus on male offspring, and it is unclear if similar metabolic benefits would have been seen in female offspring. Further, as our rats were supplemented with fish oil and not a purified n-3 PUFA concentrate, it is possible that chemical components other than the n-3 PUFAs could have had important biological effects. Notably, there are two aspects of this study that are difficult to translate to humans. First, fish oil supplementation was started immediately after mating, and thus conception. In humans, the timing of conception is mostly unknown, so that starting supplementation at this point would be difficult to achieve, unless women intending to become pregnant took supplemental fish oil. Second, while the dose of fish oil used was similar to those of previous rodent studies^25^, it is difficult to convert it to an equivalent human dose^56^. Based on surface area, it equates to approximately 30 ml in humans, which would be unacceptable to many women. Further, women wishing to take an equivalent dose of n-3 PUFAs would be seriously hindered by their inability to access fresh unoxidised supplements such as the one used in this study.

Future studies using this model of maternal high-fat diet during pregnancy should specifically assess the effects of the n-3 PUFA intervention on a number of aspects: i) maternal glucose and lipid metabolism in pregnancy; ii) on milk quality; iii) whether there is additional benefit of supplementation with fish oil during lactation; iv) whether there are alterations in gene expression and epigenetic regulation in liver, skeletal muscle and adipose tissue; v) the phenotype of female offspring. Further, a specific assessment of leptin resistance should be used, to clarify the effect of fish oil supplementation in the context of normal pregnancy.

In summary, this study showed that unoxidised fish oil supplementation during pregnancy in rat dams who were fed a high-fat diet led to a long-term beneficial effect, preventing insulin resistance in adult offspring. Thus, if these findings are translated to humans, unoxidised n-3 PUFA rich oils could have a beneficial role in mitigating adverse metabolic programming in the offspring of women who are obese during pregnancy.

## References

[1] Ng M (2014). Global, regional, and national prevalence of overweight and obesity in children and adults during 1980–2013: a systematic analysis for the Global Burden of Disease Study 2013. Lancet.

[2] Moody, A. Chapter 10: adult anthropometric measures, overweight and obesity. In *Health Survey for England 2013* (Health and Social Care Information Centre, 2014).

[3] Flegal K, Carroll M, Ogden C, Curtin L (2010). Prevalence and trends in obesity among US adults, 1999-2008. JAMA.

[4] Schneider S, Bock C, Wetzel M, Maul H, Loerbroks A (2012). The prevalence of gestational diabetes in advanced economies. J Perinat Med.

[5] Drake AJ, Reynolds RM (2010). Impact of maternal obesity on offspring obesity and cardiometabolic disease risk. Reproduction.

[6] Derraik JG, Ahlsson F, Diderholm B, Lundgren M (2015). Obesity rates in two generations of Swedish women entering pregnancy, and associated obesity risk among adult daughters. Sci Rep.

[7] Catalano PM (2009). Perinatal risk factors for childhood obesity and metabolic dysregulation. Am J Clin Nutr.

[8] Mingrone G (2008). Influence of maternal obesity on insulin sensitivity and secretion in offspring. Diabetes Care.

[9] Derraik JGB, Ayyavoo A, Hofman PL, Biggs JB, Cutfield WS (2014). Increasing maternal pre-pregnancy BMI is associated with reduced insulin sensitivity and increased blood pressure in their children. Clin Endocrinol.

[10] Perng W, Gillman MW, Mantzoros CS, Oken E (2014). A prospective study of maternal prenatal weight and offspring cardiometabolic health in midchildhood. Ann Epidemiol.

[11] Boney CM, Verma A, Tucker R, Vohr BR (2005). Metabolic syndrome in childhood: association with birth weight, maternal obesity, and gestational diabetes mellitus. Pediatrics.

[12] Catalano PM, Kirwan JP, Haugel-de Mouzon S, King J (2003). Gestational diabetes and insulin resistance: role in short-and long-term implications for mother and fetus. J Nutr.

[13] Ruchat S-M, Hivert M-F, Bouchard L (2013). Epigenetic programming of obesity and diabetes by in utero exposure to gestational diabetes mellitus. Nutr Rev.

[14] Dabelea D, Crume T (2011). Maternal environment and the transgenerational cycle of obesity and diabetes. Diabetes.

[15] DeFronzo RA, Ferrannini E (1991). Insulin resistance: a multifaceted syndrome responsible for NIDDM, obesity, hypertension, dyslipidemia, and atherosclerotic cardiovascular disease. Diabetes Care.

[16] Howie GJ, Sloboda DM, Kamal T, Vickers MH (2009). Maternal nutritional history predicts obesity in adult offspring independent of postnatal diet. J Physiol.

[17] Desai M (2014). Maternal obesity and high-fat diet program offspring metabolic syndrome. Am J Obstet Gynecol.

[18] Srinivasan M, Katewa SD, Palaniyappan A, Pandya JD, Patel MS (2006). Maternal high-fat diet consumption results in fetal malprogramming predisposing to the onset of metabolic syndrome-like phenotype in adulthood. Am J Physiol Endocrinol Metab.

[19] Kirk SL (2009). Maternal obesity induced by diet in rats permanently influences central processes regulating food intake in offspring. PLoS ONE.

[20] Rajia S, Chen H, Morris MJ (2010). Maternal overnutrition impacts offspring adiposity and brain appetite markers‐modulation by postweaning diet. J Neuroendocrinol.

[21] Muhlhausler BS, Ailhaud GP (2013). Omega-6 polyunsaturated fatty acids and the early origins of obesity. Curr Opin Endocrinol Diabetes Obes.

[22] Muhlhausler B, Gibson R, Makrides M (2011). The effect of maternal omega-3 long-chain polyunsaturated fatty acid (n-3 LCPUFA) supplementation during pregnancy and/or lactation on body fat mass in the offspring: a systematic review of animal studies. Prostaglandins Leukot Essent Fatty Acids.

[23] Mathai ML (2004). Does perinatal ω‐3 polyunsaturated fatty acid deficiency increase appetite signaling?. Obesity.

[24] Massiera F (2003). Arachidonic acid and prostacyclin signaling promote adipose tissue development a human health concern?. J Lipid Res.

[25] Oh DY (2010). GPR120 is an omega-3 fatty acid receptor mediating potent anti-inflammatory and insulin-sensitizing effects. Cell.

[26] Storlien LH (1987). Fish oil prevents insulin resistance induced by high-fat feeding in rats. Science.

[27] Derosa G (2012). Effects of n-3 PUFAs on postprandial variation of metalloproteinases, and inflammatory and insulin resistance parameters in dyslipidemic patients: evaluation with euglycemic clamp and oral fat load. J Clin Lipidol.

[28] Derosa G, Cicero AF, D’Angelo A, Borghi C, Maffioli P (2016). Effects of n‐3 pufas on fasting plasma glucose and insulin resistance in patients with impaired fasting glucose or impaired glucose tolerance. Biofactors.

[29] Haghiac M (2015). Dietary omega-3 fatty acid supplementation reduces inflammation in obese pregnant women: a randomized double-blind controlled clinical trial. PLoS ONE.

[30] Albert BB (2015). Fish oil supplements in New Zealand are highly oxidised and do not meet label content of n-3 PUFA. Sci Rep.

[31] Global Organisation for EPA and DHA Omega-3. *GOED Voluntary Monograph Version 5*, http://www.goedomega3.com/index.php/files/download/350 (2015).

[32] Albert BB (2016). Oxidised fish oil in rat pregnancy causes high newborn mortality and increases maternal insulin resistance. Am J Physiol Regul Integr Comp Physiol.

[33] Gray C, Li M, Reynolds CM, Vickers MH (2013). Pre-weaning growth hormone treatment reverses hypertension and endothelial dysfunction in adult male offspring of mothers undernourished during pregnancy. PLoS ONE.

[34] Matsuda M, DeFronzo RA (1999). Insulin sensitivity indices obtained from oral glucose tolerance testing: comparison with the euglycemic insulin clamp. Diabetes Care.

[35] Lorenzo C, Haffner SM, Stancakova A, Laakso M (2010). Relation of direct and surrogate measures of insulin resistance to cardiovascular risk factors in nondiabetic Finnish offspring of type 2 diabetic individuals. J Clin Endocrinol Metab.

[36] Trung VN, Yamamoto H, Murata S, Kuwahara A, Tani T (2014). Ileal glucose infusion leads to increased insulin sensitivity and decreased blood glucose levels in wistar rats. J Invest Surg.

[37] Cacho J, Sevillano J, de Castro J, Herrera E, Ramos MP (2008). Validation of simple indexes to assess insulin sensitivity during pregnancy in Wistar and Sprague-Dawley rats. Am J Physiol Endocrinol Metab.

[38] Hanebutt FL, Demmelmair H, Schiessl B, Larqué E, Koletzko B (2008). Long-chain polyunsaturated fatty acid (LC-PUFA) transfer across the placenta. Clin Nutr.

[39] Desvergne B, Wahli W (1999). Peroxisome proliferator-activated receptors: nuclear control of metabolism. Endocr Rev.

[40] Barak Y (1999). PPARγ is required for placental, cardiac, and adipose tissue development. Mol Cell.

[41] Myers MG, Cowley MA, Münzberg H (2008). Mechanisms of leptin action and leptin resistance. Annu Rev Physiol.

[42] Woodman RJ (2002). Effects of purified eicosapentaenoic and docosahexaenoic acids on glycemic control, blood pressure, and serum lipids in type 2 diabetic patients with treated hypertension. Am J Clin Nutr.

[43] Rizza S (2009). Fish oil supplementation improves endothelial function in normoglycemic offspring of patients with type 2 diabetes. Atherosclerosis.

[44] Giacco R (2007). Fish oil, insulin sensitivity, insulin secretion and glucose tolerance in healthy people: is there any effect of fish oil supplementation in relation to the type of background diet and habitual dietary intake of n-6 and n-3 fatty acids?. Nutr Metab Cardiovasc Dis.

[45] Rosenfeld CS (2003). Striking variation in the sex ratio of pups born to mice according to whether maternal diet is high in fat or carbohydrate. PNAS.

[46] Dama MS, Singh NMP, Rajender S (2011). High fat diet prevents over-crowding induced decrease of sex ratio in mice. PLoS ONE.

[47] Gray C (2013). Maternal fructose and/or salt intake and reproductive outcome in the rat: Effects on growth, fertility, sex ratio, and birth order. Biol Reprod.

[48] Bird E, Contreras RJ (1986). Maternal dietary sodium chloride levels affect the sex ratio in rat litters. Physiol Behav.

[49] Tataranni PA, Ortega E (2005). A burning question: Does an adipokine-induced activation of the immune system mediate the effect of overnutrition on type 2 diabetes?. Diabetes.

[50] Weisberg SP (2003). Obesity is associated with macrophage accumulation in adipose tissue. J Clin Invest.

[51] Bays H, Mandarino L, DeFronzo RA (2004). Role of the adipocyte, free fatty acids, and ectopic fat in pathogenesis of type 2 diabetes mellitus: peroxisomal proliferator-activated receptor agonists provide a rational therapeutic approach. J Clin Endocrinol Metab.

[52] Shi H (2006). TLR4 links innate immunity and fatty acid–induced insulin resistance. J Clin Invest.

[53] Guelinckx I, Devlieger R, Mullie P, Vansant G (2010). Effect of lifestyle intervention on dietary habits, physical activity, and gestational weight gain in obese pregnant women: a randomized controlled trial. Am J Clin Nutr.

[54] Muhlhausler BS, Gibson RA, Makrides M (2010). Effect of long-chain polyunsaturated fatty acid supplementation during pregnancy or lactation on infant and child body composition: a systematic review. Am J Clin Nutr.

[55] Rytter D (2011). Intake of fish oil during pregnancy and adiposity in 19-y-old offspring: follow-up on a randomized controlled trial. Am J Clin Nutr.

[56] Reagan-Shaw S, Nihal M, Ahmad N (2008). Dose translation from animal to human studies revisited. FASEB J.

